# Long-Term Clinical Outcome of a Surgically Treated Ameloblastoma: Over a Decade of Follow-Up and Oral Rehabilitation

**DOI:** 10.3390/dj14010039

**Published:** 2026-01-07

**Authors:** Ruxandra Elena Luca, Ciprian Ioan Roi, Alexandra Roi, Eduard Gîdea-Paraschivescu

**Affiliations:** 1 University Clinic of Oral Rehabilitation and Dental Emergencies, Faculty of Dentistry, “Victor Babes” University of Medicine and Pharmacy, Eftimie Murgu Square No. 2, 300041 Timisoara, Romania; 2 Interdisciplinary Research Center for Dental Medical Research, Lasers and Innovative Technologies, Revolutiei 1989 Avenue No. 9, 300070 Timisoara, Romania; 3 University Clinic of Anesthesiology and Oral Surgery, Research Center of Dento-Alveolar Surgery, Anesthesia and Sedation in Dental Medicine, “Victor Babes” University of Medicine and Pharmacy, Eftimie Murgu Square No. 2, 300041 Timisoara, Romania; ciprian.roi@umft.ro; 4 University Clinic of Oral Pathology, Multidisciplinary Center for Research, Evaluation, Diagnosis and Therapies 11 in Oral Medicine, “Victor Babes” University of Medicine and Pharmacy, Eftimie Murgu Square No. 2, 300041 Timisoara, Romania; 5 Oral and Maxillofacial Surgery Clinic, Emergency University Municipal Hospital Timisoara, Take Ionescu Bd., No. 5, 300062 Timisoara, Romania; eduardparaschivescu@gmail.com

**Keywords:** ameloblastoma, dental implants, reconstructive surgery

## Abstract

**Background**: Ameloblastomas account for roughly 1% of all jaw tumours and cysts, typically manifesting as slow-growing, painless swellings that expand both buccal and lingual cortical plates and may infiltrate adjacent soft tissue, often leading to a delayed diagnosis. These benign tumours, characterized by local invasiveness, originate from epithelial tissues and may develop from dental lamina cell rests, the enamel apparatus, the epithelial lining of odontogenic cysts, or basal epithelial cells of the oral mucosa. **Methods**: This paper aims to describe the comprehensive and interdisciplinary management of an extensive ameloblastoma in a 16-year-old patient, emphasizing the diagnostic challenges, surgical resection, reconstructive procedures, and subsequent oral rehabilitation. **Results**: At the eleven-year follow-up, clinical and radiographic examinations showed no signs of tumour recurrence. The patient presented no symptoms, indicating neither pain nor functional impairment. The prosthetic rehabilitation utilizing implant-supported fixed restorations was successfully completed, resulting in satisfactory masticatory function and aesthetics. This case adds to the existing evidence on the management of extensive ameloblastomas by demonstrating successful long-term outcomes following interdisciplinary surgical reconstruction and rehabilitation. **Conclusions**: The presented case highlights the complexity of restoring the lost tissues and functions, as well as the long-term clinical, functional, and aesthetic outcomes over an eleven-years follow-up period.

## 1. Introduction

Ameloblastoma is a prevalent benign odontogenic neoplasm, representing about 1% of all oral tumours and cysts in the jaw. About 80% of ameloblastomas are located in the mandible, predominantly in the region of the third molar [1]. It frequently manifests as a gradually enlarging, asymptomatic mass that leads to the enlargement of the cortical bone, perforation of the lingual or buccal plates, and infiltration of soft tissue [2,3]. The symptoms are clinically non-specific. They include asymptomatic swelling that may result in significant facial deformities, dental loss, ulceration, or periodontal issues. The radiographic lesions present as unilocular or multilocular radiolucencies showing a “soap-bubble” or “honeycombed” appearance. In many instances, ameloblastomas manifest as a well-defined radiolucency encircling the crown of an unerupted tooth, resembling a dentigerous cyst [4].

The precise etiology of ameloblastoma remains unidentified. Nevertheless, evidence indicates that it originates from residual odontogenic epithelium (the cells responsible for tooth formation) that remains in the jawbones [5]. Ameloblastoma may arise from remnants of the dental lamina, a growing enamel organ, the epithelial lining of an odontogenic cyst, or the basal cells of the oral mucosa [6,7]. Evidence suggests that genetic mutations and anomalies may contribute to the development of ameloblastoma, although the precise genes implicated remain unidentified [5].

As per the WHO classification from 2022, there are five primary varieties of ameloblastoma: unicystic ameloblastoma, extraosseous or peripheral ameloblastoma, conventional ameloblastoma, adenoid ameloblastoma, and metastasizing ameloblastoma. The most prevalent type is the conventional ameloblastoma, with the unicystic type being the second most common [8]. Conventional ameloblastoma is a locally invasive tumour that grows slowly. Certain ameloblastomas develop into enormous tumours that provoke the destruction of the surrounding tissues. In contrast, unicystic ameloblastoma is more common in younger populations and has a reduced recurrence rate and less aggressive nature. The tumour may either be a de novo tumour or one that develops from an odontogenic cyst. Solid and unicystic ameloblastoma are frequently observed in the mandible, particularly in the molar-ramus region [9]. The solid or multicystic variant is more aggressive and necessitates a more drastic therapy compared to other forms, exhibiting a considerably greater recurrence rate.

Prior to the recent update to the World Health Organisation (WHO) Classification of Head and Neck Tumours, as outlined by Vered and Wright [10], unicystic ameloblastoma (UA) was categorized into three histological variants: luminal, intraluminal, and mural. In the current classification, the mural subtype remains within the unicystic ameloblastoma category for treatment planning, illustrating continuity in clinical considerations while standardizing terminology. Surgical management of ameloblastoma continues to depend on tumour type and extent. For unicystic ameloblastoma, treatment strategies range from conservative procedures—including enucleation, enucleation with curettage, and marsupialization or decompression prior to enucleation—to more radical approaches such as segmental, marginal, partial, or total resections [7,11].

Since the treatment of ameloblastoma typically involves surgical excision along with surrounding tissues, as the tumour is characterized by its aggressive, asymptomatic nature and slow growth, coupled with a high recurrence rate [1], when selecting surgical treatment, it is essential to evaluate multiple aspects, including the tumour type, anatomical location, disease extent, histological and radiographic characteristics, as well as the patient’s age and cooperation [12]. The resection of the mandible, encompassing the condyle and extensive anterior region, leads to significant aesthetic, functional, and reconstructive challenges in developing young individuals [12]. An interdisciplinary approach has been suggested for the reconstruction of the dentofacial region in order to address the aesthetic and functional limitations that result from surgical excision. In this interdisciplinary approach, orthodontic treatment can play a critical role in establishing an optimal occlusal relationship and sufficient space to facilitate the successful reconstruction of the afflicted jaw region [1]. When the defect involves the coronoid and condylar processes, reconstruction of the temporomandibular joint is essential and may optimally include a patient-specific total joint prosthesis for the temporomandibular joint (TMJ). This system consists of a dual-component design, incorporating tailored fossa and mandibular components that accurately align with patient anatomy [13].

Bone changes related to edentulism typically complicate prosthetic planning, affecting both straightforward and complex rehabilitation efforts. The resection of a significant portion of the mandible can impede the establishment of a new occlusal balance and necessitates careful consideration to achieve a stable and non-iatrogenic treatment outcome [14].

Given the rare, yet extensive and aggressive nature of this type of tumour, the necessity of a treatment that prevents recurrence, and the challenges of reconstructing hard and soft tissues to provide functional and aesthetic rehabilitation for the patient, we consider that this type of pathology necessitates an interdisciplinary approach, involving a maxillofacial surgeon, orthodontist, prosthodontist, and occasionally a psychologist, as well as effective synchronisation of the treatment stages and follow-up. This paper aims to describe the comprehensive and interdisciplinary management of an extensive ameloblastoma in a 16-year-old patient, emphasizing the diagnostic challenges, surgical resection, reconstructive procedures, and subsequent oral rehabilitation. The report highlights the complexity of restoring the lost tissues and functions, as well as the long-term clinical, functional, and aesthetic outcomes over a ten-year follow-up period.

This case report has been prepared in accordance with the CARE guidelines for case reports (https://www.care-statement.org/, accessed on 4 August 2025).

## 2. Materials, Methods and Results

### 2.1. Initial Presentation

**Figure 1:**
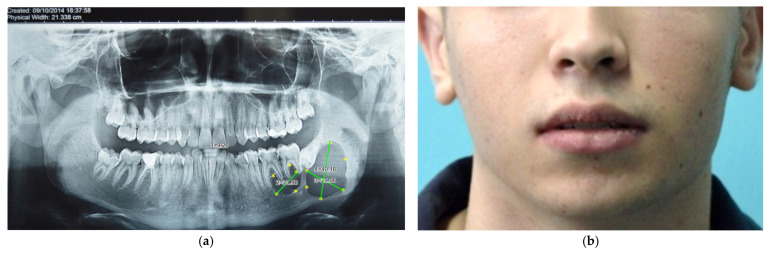
Initial orthopantomogram, with delineation of the bone lesion boundaries and corresponding image measurements (**a**), Exofacial aspect of the patient (**b**).

A 16-year-old male patient was referred to the Oral and Maxillofacial Surgery Department of Emergency Hospital Timisoara following the detection of a mandible tumor formation on orthopantomography (Figure 1a). The patient presented with pain in the left mandibular region and slight local edema (Figure 1b). The radiograph shows an extended multilocular radiolucency in the region of the left mandibular angle, involving the first and second molars, as well as the 3rd molar, which is impacted in a horizontal position. The radiograph also reveals mesial and distal coronal radiolucencies in the first molar, and mesial in the second molar, suggesting the presence of carious lesions.

**Figure 2:**
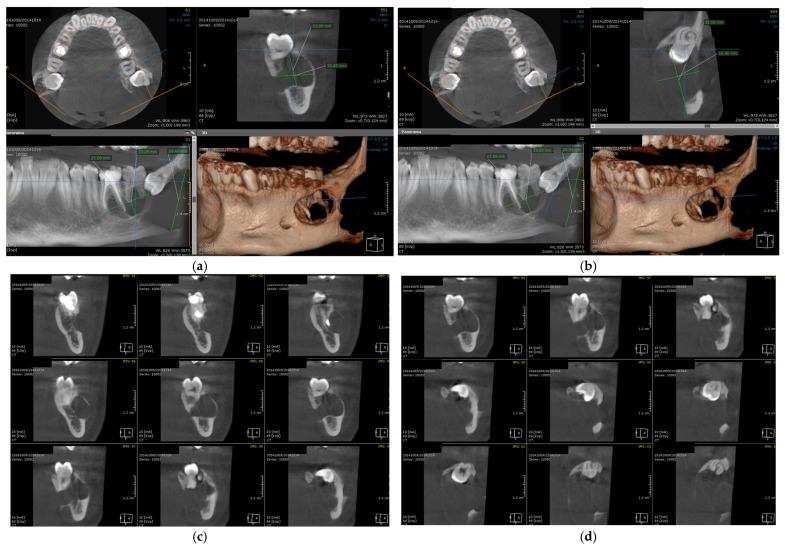
Initial CBCT, different aspects showing the extent of the tumor, in axial, sagittal, frontal and reconstruction view (**a**,**b**) and 2 mm slices in axial view (**c**,**d**).

The patient is urgently referred for a CBCT, which provides three-dimensional details regarding the extension of the tumor process: distension of the vestibular and lingual bone corticals, in the region of the left mandibular angle, which communicates with another mesially situated unilocular radiolucency, impeding the roots of the first and second left mandibular molars, up to the furcation area (Figure 2). Even this second lesion appears to deform and perforate both corticals.

### 2.2. Surgical Step 1

The patient is admitted to the Oral and Maxillofacial Surgery Department of Emergency Hospital Timisoara, with the diagnosis of bilocular cystic tumor formation in the left mandibular angle, impacted third molar, allergy to Ciprofloxacin, with pain and masticatory disorders as main complaints.

**Figure 3:**
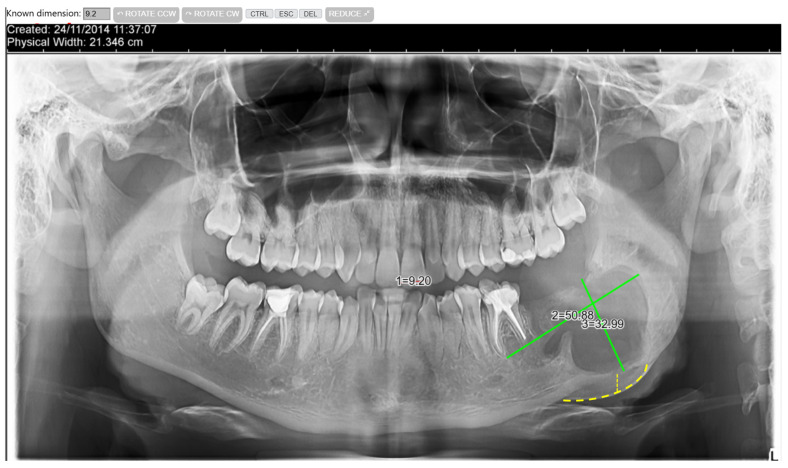
Radiological aspect after extraction of 3.7, 3.8 and enucleation of the cyst, with delineation of the bone lesion boundaries and corresponding image measurements (the solid green lines represent measurements in two planes of the lesion, while the dotted yellow lines suggest a third dimension of the lesion, resulting from the expansion and deformation of the bone contour).

Following the clinical and paraclinical examination, the decision is made to perform an excisional biopsy, completed by the removal of the involved teeth. Thus, under general anesthesia (oro-tracheal intubation) supplemented with local anesthesia, the teeth 3.7 and 3.8 are extracted, along with the removal of the cystic membrane (enucleation), with preservation of the inferior alveolar nerve, which appears to be dislocated inferiorly and lingually (Figure 3). The specimens are sent for histopathological examination, and the patient undergoes antibiotic and anti-inflammatory treatment (Clindamycin 600 mg twice daily for 7 days/Ibuprofen 400 mg).

### 2.3. Histopathological Diagnosis

The histopathological analysis reveals the following aspects:

- Tissue fragments containing a cellular proliferation consisting of odontogenic epithelium with columnar cells with “reverse polarization” nuclei (nuclei oriented away from the basal lamina) and epithelial cells with an appearance similar to the stellate reticulum of the enamel organ organized in solid areas, islands, cords, cystic areas in a richly collagenized fibrous conjunctival stroma.
- Random, there are aspects of squamous metaplasia and areas in which the tumor cells have fine granular cytoplasm present.
- Associated, an inflammatory lympho-plasmacytic infiltrate and macrophages with foamy cytoplasm are observed.

**Figure 4:**
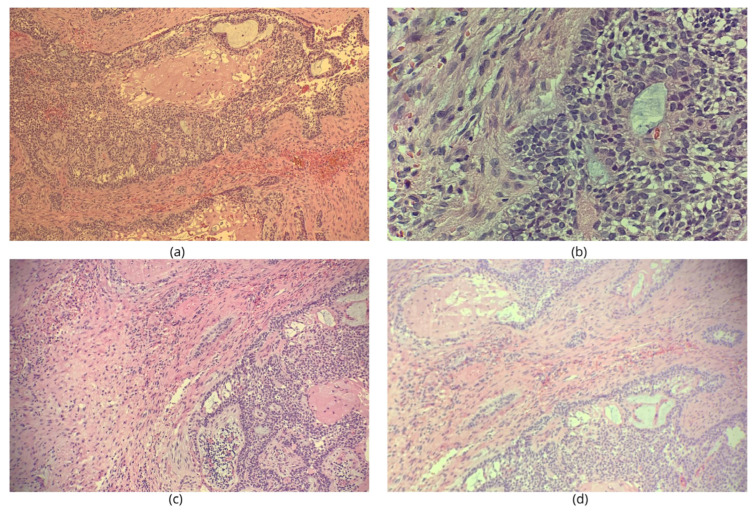
Microscopic aspects of plexiform ameloblastoma in hematoxylin-eosin staining (**a**–**d**).

Conclusion: ameloblastoma with conventional areas, follicular type, plexiform type, acanthomatous type and with granular cells (Figure 4).

### 2.4. Surgical Step 2

**Figure 5:**
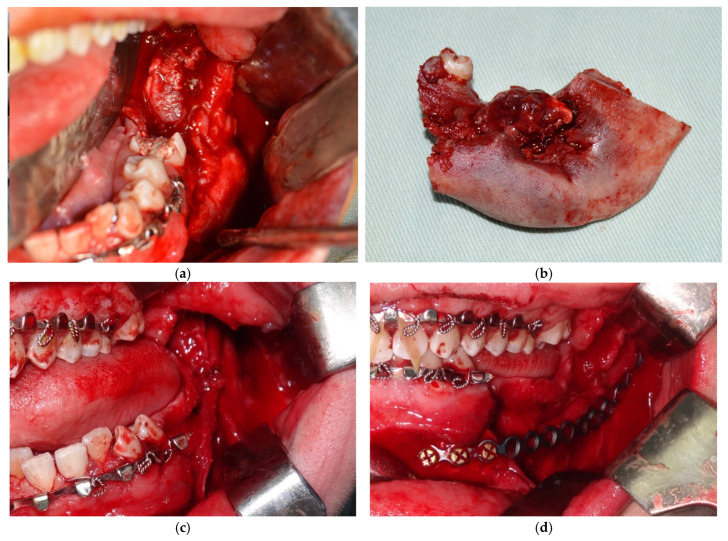
Intraoperative aspects of the second surgical phase: initial aspect of the tumor (**a**), mandibular resected piece (**b**), preservation of the inferior alveolar nerve (**c**), fixation using a titanium plate with 16 holes and 5 screws (**d**).

**Figure 6:**
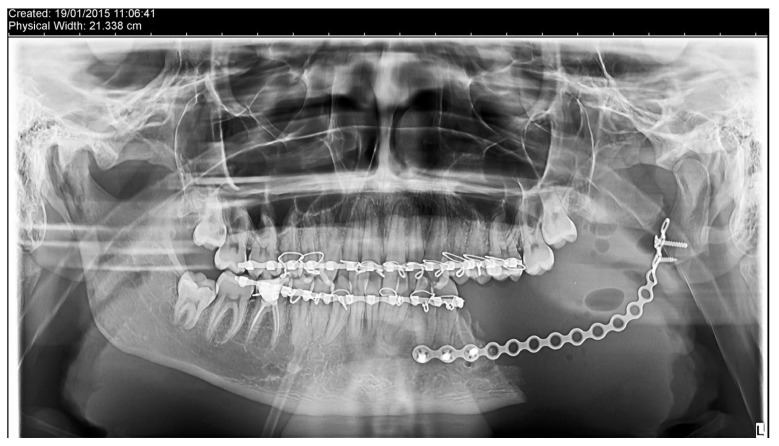
Postoperative aspects of the reconstruction utilizing a titanium plate with 16 holes and 5 screws.

Based on the histopathological analysis, a decision was made to proceed with a second surgical intervention involving partial mandibular resection and fixation of the two fragments using a flexible titanium plate with 16 holes and 5 screws, along with a biopsy. Consequently, general anesthesia (naso-tracheal intubation) was administered, supplemented with local anesthesia, after which a flap was raised from the mesial aspect of the second premolar to one cm posterior to the retromolar triangle, along the anterior margin of the ascending branch of the mandible. The underlying bone was exposed and the partial mandibular resection was performed extending from the distal aspect of the second premolar to approximately 1 cm under the sigmoid notch (in vertical and horizontal respects). The inferior alveolar nerve was identified lingually to the resected specimen and again, preserved (Figure 5 and Figure 6). Hemostasis was achieved through cauterization and the use of TachoSil (Takeda Austria GmbH, Linz, Austria). The mandible was placed in the correct occlusal relationship and stabilized through an elastic intermaxillary fixation. The two mandibular fragments were stabilized using a flexible plate with 16 holes and 5 screws (Stryker, Portage, MI, USA), which was previously bent to respect the contour of the resected part of the mandible. Prior to plate fixation, the position of the mandibular condyle was further checked via ecographic evaluation. The surgeon decided to position the plate about 1.2 cm above the mandible’s inferior border, anticipating a reconstruction with an autologous bone graft stabilised by a lower plate.

The specimens were submitted for histopathological examination, and the patient received antibiotic and anti-inflammatory therapy (Clindamycin 600 mg/Ibuprofen 400 mg). Following the operation, a naso-gastric tube was inserted and maintained for 7 days, while tight elastic intermaxillary fixation was installed for 3 weeks. The recommendations for intermaxillary fixation included maintaining excellent oral hygiene, with light chlorhexidine rinses, consuming soft foods (with a straw, following the removal of the nasogastric tube), and avoiding contact sports to prevent local injuries.

Following the surgical intervention, the patient experienced paresthesia within the innervation area of the inferior alveolar nerve for a duration of 4 to 6 months, after which the sensitivity was fully recovered.

### 2.5. Surgical Step 3

**Figure 7:**
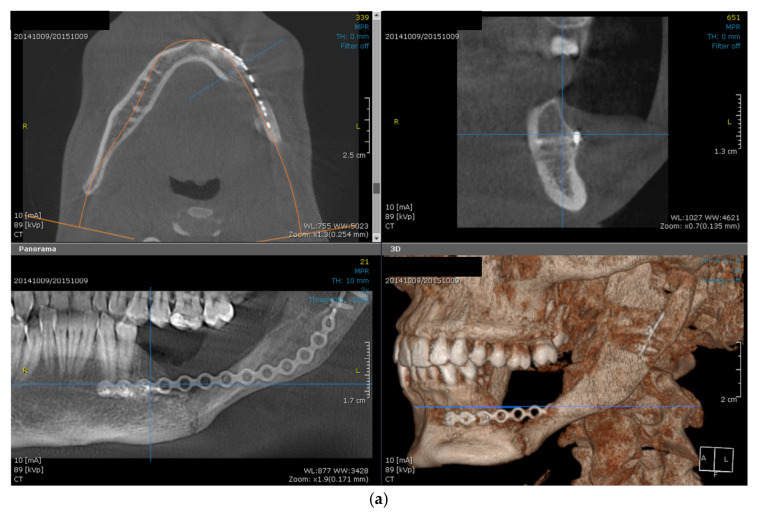

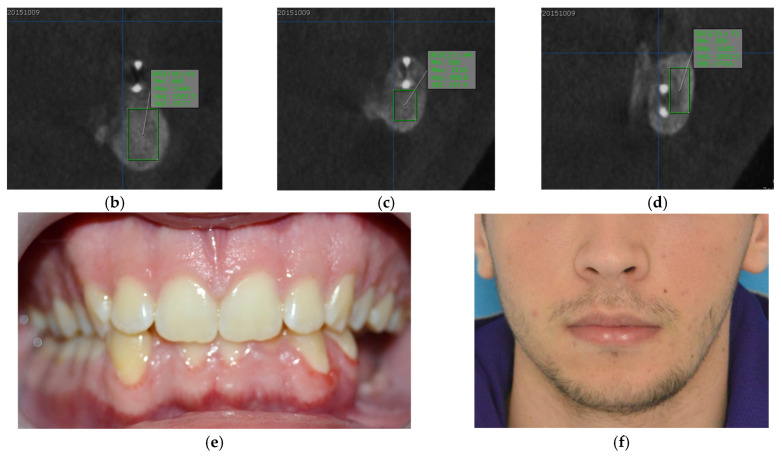
Preoperative aspect of the mandibular spontaneous healing at 10 months after mandibular resection and reconstruction using a titanium plate with 16 holes and 5 screws, in axial, sagittal, frontal and reconstruction view (**a**); different measurements of the bone density using Hounsfield Units (**b**–**d**); intraoral aspect (**e**); exooral aspect, showing no facial asymmetry (**f**).

**Figure 8:**
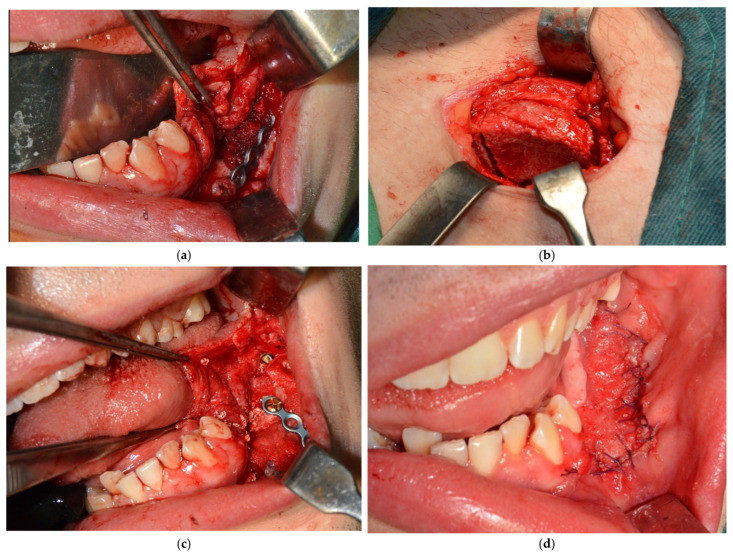
Intraoperative aspect of the mandibular reconstruction: initial view, after reflection of the flap (**a**), iliac crest prelevation (**b**), autologous bone graft, together with bovine xenograft and collagen membrane in place (**c**), intraoral aspect of the suture (**d**).

The control CBCT conducted at 10 months after the resective procedure reveals a very good bone formation (quantitatively and qualitatively—Figure 7b–d), all along the fixation plate, demonstrating a very good periosteal osteogenic activity, which, although understandable in young patients, is not yet seen in all cases of similar defects, with comparable outcomes [15,16,17]. The assessment of bone healing revealed that, although significant, the volume achieved through spontaneous healing does not meet the standards required for optimal prosthetic rehabilitation (Figure 7). Therefore, a decision was reached to undertake a third surgical intervention, this time for alveolar ridge reconstruction using iliac crest, particulate xenograft, and collagen membrane. The reconstructive procedure was conducted by two surgical teams: one responsible for preparing the receptor site and the other for harvesting the graft from the donor site at the iliac crest. Subsequently, general anesthesia (oro-tracheal intubation) was administered, enhanced with local anesthesia. The newly formed bone was then exposed via a flap similar to that used in the prior surgery, with a limited posterior extension. The receptor site remains protected with moistened gauzes while the second team finalizes the harvesting of the iliac crest graft, which includes both cortical and cancellous bone (Figure 8). The donor site is sutured in layers following the achievement of adequate local hemostasis. The graft was meticulously cleansed of all soft tissue attachments and shaped to match the dimensions of the defect. The newly formed bone was perforated to induce bleeding, and fixation of the iliac crest was achieved using a vertically positioned plate with four holes and two screws, serving as a contour scaffold for the intended bone reconstruction. To completely address the defect, a combination of bovine bone and harvested autologous cancellous bone was utilized, and the entire bone reconstruction was protected with a collagen membrane. Postoperatively, the patient receives antibiotic and anti-inflammatory therapy (Clindamycin 600 mg/Ibuprofen 400 mg).

The patient underwent psychological counseling before and after the resective surgical treatment.

One-year post-reconstruction, a minimal surgical procedure was necessary, to remove the fixation plate and screws due to the exposure of the upper screw of the autologous graft fixation.

### 2.6. Postoperative Evolution

**Figure 9:**
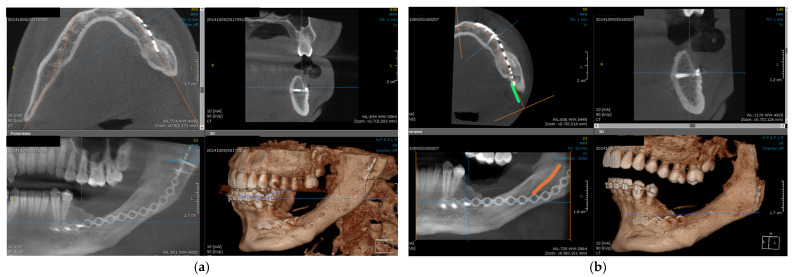
Postoperative aspect of the mandibular reconstruction at 18 months (**a**), and 3 years respectively (**b**).

Given the case’s evolution, the patient’s age, and psychological factors associated with the diagnosis and interventions, a decision is made, in collaboration with the patient’s family, to monitor the case for a set period of time, with implant-supported prosthetic rehabilitation scheduled for a later date. Thus, Figure 9 depicts the postoperative situation 18 months after reconstructive surgery, and three years later. Considering the existing positional disorders of the teeth, the patient is referred for orthodontic treatment. Before initiating the prosthetic rehabilitation stage, since dental migrations in quadrants 2 and 3 were observed, the orthodontic treatment was instituted, which was maintained until provisional prosthetics was fixed.

### 2.7. Implant Insertion and Guided Bone Regeneration

**Figure 10:**
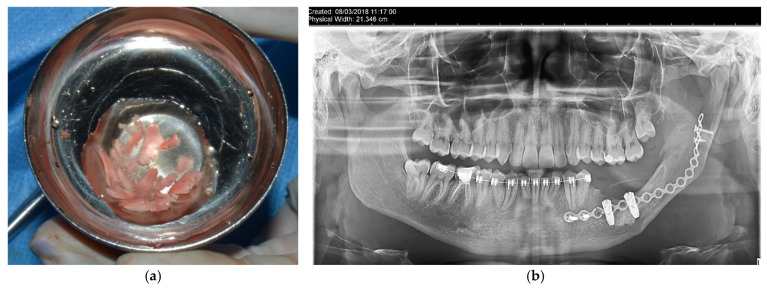
Intraoperative aspect of the bony chips used for guided bone regeneration (**a**), postoperative radiological aspect of the mandible with the implants inserted (**b**).

Considering the positive evolution of the case, as well as the psycho-emotional status of the patient, who has already become a young adult, it is decided to continue with the implant-prosthetic phase; hence, two bone level dental implants (Dentium Superline 4.5 × 12 mm, 5 × 12 mm, Dentium Co., Ltd., Seoul, Republic of Korea) are inserted and, at the same time, guided bone regeneration with bovine xenograft and autologous chips is performed, to improve the existing bone supply. Simultaneously, one of the three mesial screws was removed, in order to prevent a conflict with the implant’ positioning (Figure 10).

### 2.8. Implant-Supported Prosthetic Restoration and Laser Vestibuloplasty

**Figure 11:**
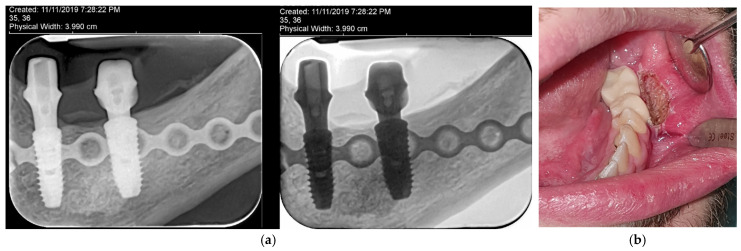
Radiological aspect of the implants and the customized titanium abutments (**a**), and aspect of the prosthetic provisional restauration in place, after diode laser vestibuloplasty (**b**).

Following the osseointegration stage, the classic prosthetic steps include the open tray impression and design of the individualised prosthetic abutments (two custom-made premill titanium abutments were fabricated in the laboratory to accommodate the increased gingival height and atypical soft tissue anatomy of the region), as well as the provisional prosthetic restorations, which will conform the peri-implant soft tissues. Since the absence of keratinised mucosa is a risk factor in implant-supported rehabilitations, a vestibuloplasty intervention is conducted (Figure 11) using a laser diode with a wavelength of 940 nm (EPIC™ X, Biolase, Foothill Ranch, CA, USA) and a 400 μm initiated tip, with the following working parameters: pulse mode 100 microseconds ON-200 microseconds OFF, peak power of 2.7 W, average power 0.9 W, pulse interval of 0.2 ms, pulse length of 0.1 ms, duty cycle of 30%.

**Figure 12:**
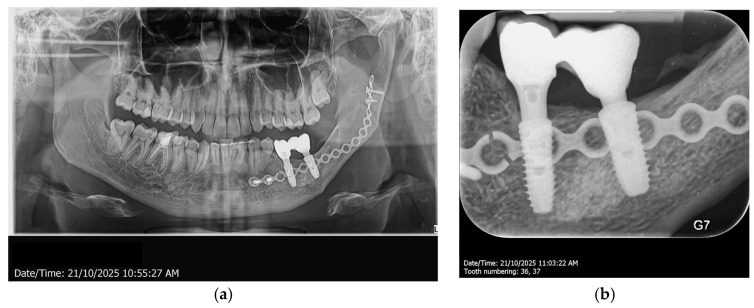
Radiological aspect of the implants, the customized titanium abutments and the zirconia restorations, 6 years in function, panoramic view (**a**) and retro-alveolar view (**b**).

Eleven years post-initial intervention, the routine examination indicates a steady bone level, demonstrating effective integration of the implants and implant-supported prosthetic restorations (Figure 12).

**Figure 13:**
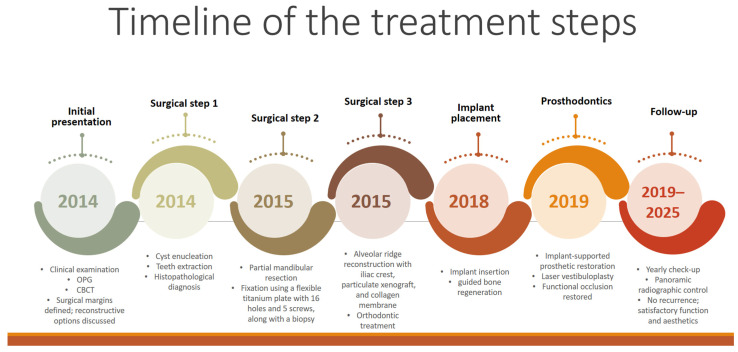
Timeline of the treatments steps: therapy stages and a summary of the most important information.

The complete progression of the patient, encompassing each therapeutic stage and summarising the essential details, is depicted in the figure below (Figure 13).

## 3. Discussion

### 3.1. Radical Resection or Conservative Management

Despite being benign and slow-growing, ameloblastomas demonstrate locally damaging characteristics and possess a high recurrence rate [18]. Numerous studies address the necessity for radical treatment in these pathologies. While we do not aim to present these studies in detail, their findings are incorporated in a comparative manner to contextualize and strengthen the interpretation of our own case.

Surgical interventions for ameloblastoma range from simple enucleation, with or without bony curettage, to radical excision. The infiltration of these tumours extends beyond macroscopic and radiological boundaries, necessitating the establishment of safety margins to avert recurrences. Consequently, many authors consider that tumour enucleation and curettage can lead to a significant risk of recurrence and an elevated likelihood of fractures, attributable to the preservation of compromised and/or weakened bony structures [18,19,20,21]. Another important aspect to take into consideration is that of the possible undetectable microscopic dissemination, particularly within the central cancellous bone; hence, radical surgical excision emerges as the sole treatment option with a satisfactory curative rate for both primary and recurrent ameloblastomas [18]. A notable case report describes the recurrence of ameloblastoma in the soft tissue, representing a rare complication [3]. The authors report the case of a 53-year-old patient, who, 24 years prior, underwent enucleation and curettage in the angle of the mandible, which was diagnosed histopathologically as ameloblastoma. After 5 years, a subsequent surgical intervention (partial resection with safety margins) was conducted due to a recurrence in the right body, ramus, and angle, which was significantly large. After another 13 years, an asymptomatic lesion was observed in the right buccal mucosa, characterized by a smooth surface, firm rubbery texture, normal color, and a sessile shape, which was treated through excisional biopsy and the diagnosis was of ameloblastoma. Therefore, there are circumstances in which this benign tumour may exhibit aggressive behaviour, a phenomenon that can be attributed to the anatomical-pathological subtype, the existence of neoplastic cells, or incomplete extirpation.

Previous discussions have highlighted the significance of extensive surgical resection in reducing recurrence risk, as evidenced by a randomised trial with 48 participants [22,23]. Of the 48 cases, eleven underwent radical resection, with no instances of recurrence observed. Of the 37 patients initially treated with conservative resection, 22 experienced recurrences. Of the 22 patients, 16 underwent conservative secondary resections, leading to recurrence in 6 cases. The authors demonstrated that initial radical resection is superior to conservative management, a conclusion that aligns with—and further supports—the treatment approach adopted in our case.

A different investigation performed a retrospective analysis of individuals with mandibular ameloblastoma to assess the management of recurrent ameloblastoma. The collected data encompassed age, gender, tumour site, histological results, initial therapy, recurrence count, year of initial diagnosis, recurrence treatment type, reconstruction, and follow-up. Nine individuals (29%) exhibited recurrences. Tumor recurrences manifested, on average, 32 months after the original surgical intervention. Recurrences were primarily linked to an insufficient initial therapeutic strategy and were addressed with bone excision with a safety margin of no less than 1 cm beyond the radiographically discernible boundaries [21], an approach consistent with the rationale behind the surgical plan implemented in our case.

Another study examining risk factors for relapse predisposition was conducted by a team of researchers from the Netherlands, who analysed 44 cases of ameloblastoma treated over a span of 40 years. In their study, of the 28 patients treated with enucleation, 17 experienced relapses, with no established correlation to an anatomotopological form or patient age; in 6 of the 17 relapses, the recurrent form differed from the initial tumour. The recurrence of some unicystic ameloblastomas can likely be attributed to the fragmentation that occurs during the excision of the original tumor. The authors also highlight the challenges related to diagnosing some subtypes of ameloblastoma: certain portions of the epithelial lining in a (uni)cystic ameloblastoma may not exhibit the characteristic features of an ameloblastoma; a biopsy taken from a primary unicystic ameloblastoma or from a recurrent, solid/multicystic ameloblastoma may not consistently reveal the usual characteristics of ameloblastoma, which could lead to an underdiagnosis and subsequently result in inappropriate management [24]. In 2014, an extensive research conducted in Australia, on 42 patients with ameloblastoma, reported that their findings align with existing literature advocating for radical surgery in the management of solid/multicystic and type 3 unicystic tumours [25]. In a more recent comprehensive systemic review conducted by a team of researchers from India, the primary factors contributing to recurrence included multilocular ameloblastomas, follicular histopathology, and conservative treatment approaches [26].

Consulting numerous similar studies, it seems that the therapeutic option of choice remains radical, but as highlighted in many studies, the patient’s age must also be taken into account, in conjunction with the extent of the future bone defect and the affected functions. Many studies have consistently demonstrated that conservative approaches—such as enucleation and curettage—are associated with a substantially higher risk of recurrence, largely due to the infiltrative and often microscopically extensive nature of ameloblastomas. As reported in both retrospective analyses and long-term follow-ups, recurrence rates after conservative treatment can exceed 50%, particularly in multilocular, follicular, and solid/multicystic variants, with several authors highlighting the limitations of relying solely on radiographic margins due to the potential for undetectable microscopic extension. Randomized and observational data further support this view and similar findings have been documented in other cohorts, where insufficient primary treatment and incomplete tumoral extirpation were identified as the predominant causes of recurrence. Against this background, the resective approach adopted in the present case aligns with the prevailing body of literature advocating for radical excision as the treatment of choice to ensure long-term disease control. What this case contributes is an extended 11-year postoperative follow-up demonstrating not only the absence of recurrence but also the successful restoration of function and aesthetics in a young patient—a dimension that reinforces the long-term validity of radical management while addressing the additional reconstructive and rehabilitative considerations in adolescent populations.

### 3.2. Surgical Options for Bone Reconstruction

A thorough, multidisciplinary strategy that is directed by well-defined therapeutic goals addressing both anatomical reconstruction and functional rehabilitation is essential for treating extensive oral and maxillofacial defects. To enable future prosthetic rehabilitation, the primary goals include: reestablishment of the mandibular continuity through accurate bony reconstruction, while ensuring sufficient bone volume; prevention of graft resorption by ensuring proper vascularization and distribution of load, especially in cases requiring dental implant placement; and effective management of soft tissue components. On one hand, we should take into consideration that even minor facial asymmetries can significantly affect an individual’s psychosocial health and on the other side, priorities for functional recovery involve restoring effective chewing ability and clear speech through correct tongue positioning and oral competence standard [27,28].

The mandible is essential for speech, mastication, swallowing, and facial aesthetics. The interruption of continuity due to radical surgery substantially impacts function and diminishes patients’ quality of life. As a result, these procedures often necessitate difficult reconstructive surgeries to restore functionality and structural integrity. In cases of tumors necessitating mandibular resection, the use of vascularized free bone grafts for reconstruction has traditionally been regarded as the gold standard [27,29].

In our particular case, for bone reconstruction after mandibular resection, the subsequent surgical reconstruction alternatives were evaluated: the iliac crest graft or non-vascularized rib, with the drawback of diminished height in the latter case or free vascularized bone grafts (fibula free flap). The fibula free flap has established its place as the standard for surgical reconstruction of mandibular bony defects since its initial application by Hidalgo in 1989. The primary challenge of a free fibula flap in mandibular reconstruction lies in the modelling and reshaping of the fibula to reach the appropriate volume and height for subsequent dental implant rehabilitation. A crucial step involves the intra-surgical accurate modelling of the titanium plate to prevent breakage resulting from improper adjustments and reshaping [30]. Concerning the microvascularized flap, the fibula may lack sufficient height, resulting in a significant disparity between the restored segment and the occlusal plane [31,32]. In our case, the surgical decision was determined by the evidence of effective spontaneous bone healing, which guaranteed a high-quality recipient bone bed. Likewise, considering the surgeon’s prior experience with rigid reconstruction plates, which are often exposed either endo- or exo-orally, as confirmed by the specialty literature [33,34], it was decided to utilize a flexible stabilization plate.

Concerning the reconstruction alternative following bone resection, since the demand for 3D printing is on the rise and is expected to become more prevalent in surgical applications in the near future, surgical resection could be followed by reconstruction using a 3D-printed osteosynthesis prosthesis aimed at restoring both functionality to the temporomandibular joint and the mandibular bone, while also considering the patient’s aesthetics [35].

### 3.3. Oral Rehabilitation Strategies and Future Directions

The most common prosthetic rehabilitation for such patients are removable guide flange prosthesis, palatal based guidance restorations and implant-supported prosthesis [36,37]. Mandibular reconstruction following ameloblastoma involves the use of implant-supported prostheses, which are considered the most effective approach for functional and aesthetic rehabilitation. Implant-supported prostheses restore essential chewing capabilities, achieving bite forces of 80–90% compared to natural teeth, while also preserving the shape of the alveolar ridge and support facial soft tissues, which is crucial given the substantial bone loss often observed after resections for ameloblastoma [27,38,39,40,41,42]. Considering the very young age of our patient, the option of fixed implant-supported restoration was the choice.

Virtual surgical planning has transformed the approach to reconstructive surgery by enabling the creation of models, as well as cutting and drilling guides. The implementation of these has decreased operative durations and improved surgical outcomes by enhancing predictability and accuracy. This technology has been expanded to include predictive placement of endosseous dental implants, thereby facilitating prosthetic rehabilitation during primary reconstruction [43]. A recent 2023 study showcased an extensive digital workflow for managing ameloblastoma. The results suggest that CAD-CAM presurgical planning supports optimal bone recovery, maintains the vitality of the free flap, and facilitates both anatomical and functional restoration of the mandible via dental implants, significantly improving the patient’s quality of life. The timing of dental implant placement is crucial to achieve successful results for patients in need of this reconstructive approach. The benefits of surgical CAD/CAM reconstructive procedures include optimal pre-surgical planning for tumor removal and consistent surgical techniques for osteotomies regarding their location and orientation. CAD/CAM prototyping enables the modeling of the titanium plate before surgery, which lessens the stress and bending fatigue on the plate, thereby decreasing the likelihood of fractures. Furthermore, the digital fabrication of osteotomy guides ensured a strong bone-to-bone connection between the distal fibula segment and the remaining mandible, enhancing the facial symmetry following reconstruction. Digital surgical planning significantly shortens operation time, leading to decreased blood loss and a reduced risk of ischemia in the fibula flap [30].

Looking ahead, emerging digital innovations are expected to further refine these workflows. The integration of artificial intelligence for automated segmentation and implant positioning, augmented reality–assisted intraoperative navigation, and fully virtualized prosthetic-driven planning represent promising advancements that may enhance accuracy and efficiency even further. Additionally, the increasing use of intraoral and facial scanners, 3D-printed patient-specific implants, and real-time adaptation of surgical plans is likely to advance personalization in complex maxillofacial reconstruction. Together, these evolving technologies will continue to expand the capabilities and clinical impact of digital workflows in reconstructive surgery.

The fundamental goal of mandibular reconstruction is to create the osseous foundation required for endosseous implant insertion. The viability of endosseous implants in composite free flaps is critical for providing the necessary support for dental prostheses, which improves mastication, speech, and facial appearance. A systematic review was conducted to characterize the survival of implants within composite free flaps for gnathic reconstruction [44]. Significant variability existed among the included studies regarding definitions of implant survival and success. However, a pooled five-year survival rate of 94% was observed for 1328 implants placed in either a fibula free flap or deep circumflex iliac artery flap, with no statistically significant differences in survival between the groups.

Another comprehensive review regarding oral rehabilitation with dental implants in patients treated after ameloblastoma removal was conducted in 2023 and revealed valuable data. A total of 64 patients and 271 implants were examined, all of whom underwent surgical treatment for AM. The ages of the patients varied from 8 to 79 years, with a mean age of 37.3 years (SD = 16.4). Fifty-three percent of the participants were male, while 47 percent were female. The follow-up duration varied from 1 to 22 years. A reported implant survival/success rate is 98.1%. Furthermore, a majority of them were conventionally loaded (38.3%). Hybrid implant-supported fixed dentures were utilized by 53% of prosthodontists. None of the evaluated studies reported recurrences of the tumor lesion following dental implant placement, indicating the high effectiveness of the surgical treatment [45]. In conclusion, oral rehabilitation utilizing dental implants placed in free flaps for orofacial reconstruction in surgically treated patients with AM is a viable and effective treatment approach.

A recent systematic review examining health-related quality-of-life measures after various radical surgical treatments for ameloblastomas indicated improvements across several metrics, including pain, appearance, activity, swallowing, chewing, speech, mood, and anxiety. Of the 283 patients included in the studies, only 73 underwent oral rehabilitation, leading to significant improvements in chewing and diet scores [43,46].

The utilization of recombinant human morphogenetic protein as a graft material yields promising results, demonstrating favourable clinical outcomes. Certain studies indicate a successful correlation between rhBMP type 2 and bovine bone xenograft, illustrating that the implants situated in the grafted region were preserved without signs of bone resorption [47].

An additional area of interest in the clinical management of this pathology, as well as in partial mandibular resections and subsequent bone reconstructions, is the inclusion of laser therapy and its effects on both hard and soft tissues through photobiomodulation (PBM). The potential benefits include enhancing the quantity and quality of newly formed bone, supporting neurosensory recovery, and promoting faster and more comfortable healing. These expected outcomes are in agreement with findings from several PBM investigations conducted by our research group. For example, optical coherence tomography and microtomographic evaluations have shown that PBM can improve the rate and structural organization of bone regeneration in animal models [48,49]. Additional experimental work has demonstrated that PBM can enhance the biological environment of implant site preparation [50] and support more favorable osseointegration outcomes when compared with conventional surgical techniques [51]. More recently, clinical imaging–based evidence indicated that PBM may contribute to increased peri-implant bone density in human subjects [52].

When contextualized against these data, the present case shows a consistent pattern of improved postoperative evolution, particularly with respect to tissue healing and functional recovery, despite the increased clinical complexity associated with mandibular pathology and reconstruction. Although the present case did not involve sensorial impairment (only a temporary paresthesia, lasting 4–6 months), it is important to note that such complications frequently arise in similar clinical scenarios. In those situations, photobiomodulation may offer a suitable therapeutic approach, given its established benefits in promoting neural recovery and reducing inflammation.

Anticipating the recurrence of an ameloblastoma before surgery would enable the modification of the treatment strategy for each individual case. Consequently, recurrence may be reduced without increasing surgical morbidity or extending mandibular resection [53]. Other treatment modalities have been proposed by various authors who argue that immediate reconstruction following an en bloc resection with safety margins is the optimal approach for managing ameloblastomas. This method facilitates complete disease removal while simultaneously addressing patient cosmetic and functional rehabilitation within a single surgical procedure [54].

More recent methods in the diagnosis and treatment of AM are those that include immunohistochemical and molecular analyses. Although molecular diagnostics—particularly detection of BRAF V600E mutation—have emerged as a potential avenue for refining the diagnosis, prognostic assessment, and even future targeted therapies in ameloblastoma, their current clinical utility remains limited by several factors. A 2022 meta-analysis estimated the pooled prevalence of BRAF V600E mutations in ameloblastomas at approximately 70.5% [55]. While immunohistochemistry (IHC) using mutation-specific antibodies such as VE1 has shown high sensitivity and specificity compared with molecular tests in some series, the evidence base remains narrow and heterogeneous [56]. Additionally, a recent systematic review and meta-analysis comparing various IHC markers between benign ameloblastoma and malignant ameloblastic carcinoma identified only SOX2 as having consistent differential expression; for most other markers, reproducibility, inter-study concordance, and clinical significance remain uncertain [57]. Methodological variability, retrospective designs, small sample sizes, and lack of standardized protocols further hamper the generalization of findings. Therefore, in our case—which predates widespread use of these techniques in our region and for which tissue specimens were limited for molecular testing—the diagnosis and management relied on conventional histopathology, clinical, radiographic, and surgical criteria. We recognize this limitation and emphasize the need for future prospective studies with standardized molecular/IHC protocols to clarify the prognostic and therapeutic relevance of molecular markers in ameloblastoma.

## 4. Conclusions

Managing ameloblastomas requires a team of specialists who focus on both the removal of the tumor and the reconstruction process, as well as the oral rehabilitation aspects, in addition to addressing the social and emotional aspects of the illness. In our case observed over an 11-year period, surgical resection, along with bone grafting from the iliac crest and prosthetic reconstruction using implants, showed lasting functional and aesthetic stability, with no evidence of recurrence and no discomfort experienced by the patient. A continuous dedication and cooperative effort from the team, along with patients and their families, are crucial for achieving the best possible results in such complex cases.

We regard the long-term evaluation in ameloblastoma cases as essential, and its importance is consistently reinforced by evidence from multiple large retrospective studies with follow-up periods extending 10 to 30 years. These investigations collectively show that recurrence is not only time-dependent—with rates rising steadily over 5, 10, and 15 years—but is also strongly influenced by treatment modality, with radical resection repeatedly identified as the most reliable predictor of lower recurrence [58,59]. Additional factors such as the presence of impacted teeth, root resorption, tumor size, and imaging-based classification further refine recurrence risk assessment. At the same time, long-term data underscore that conservative approaches may be appropriate for selected cases such as unicystic ameloblastomas, provided that rigorous and prolonged follow-up is ensured [60,61,62]. Altogether, the consistency of these findings across diverse populations and methodological approaches highlights the necessity of extended postoperative surveillance and supports our emphasis on long-term evaluation in managing ameloblastoma.

## Data Availability

The original contributions presented in the study are included in the article; further enquiries can be directed to the corresponding authors.

## Abbreviations

The following abbreviations are used in this manuscript:

WHO	World Health Organisation
UA	unicystic ameloblastoma
TMJ	temporo-mandibular joint
AM	ameloblastoma
rhBMP	recombinant human bone morphogenetic protein
PBM	photobiomodulation
IHC	immunohistochemistry
